# Level of exclusive breastfeeding practice in remote and pastoralist community, Aysaita woreda, Afar, Ethiopia

**DOI:** 10.1186/s13006-019-0200-6

**Published:** 2019-01-29

**Authors:** Medhin Tsegaye, Dessalegn Ajema, Solomon Shiferaw, Robel Yirgu

**Affiliations:** 1 Department of Public Health, Myungsung Medical College, P.O. Box: 14972, Addis Ababa, Ethiopia; 2 Department of Public Health, Arba Minch University, P. O. Box: 21, Arba Minch, Ethiopia; 3 School of Public Health, Addis Ababa University, P.O. Box: 9086, Addis Ababa, Ethiopia

**Keywords:** Exclusive breastfeeding, Pastoralist community, Remote area, Afar, Ethiopia

## Abstract

**Background:**

In Afar, a pastoralist and remote area of Ethiopia, one in five children suffers from acute malnutrition. Investigation of the prevalence and associated factors of exclusive breastfeeding may provide insight into the current burden and nature of the problem, and offer help on how to direct prevention strategies. The aim of this study was to measure the prevalence and identify associated factors of exclusive breastfeeding (EBF) practice in Afar, Ethiopia.

**Methods:**

A community based cross-sectional study was conducted with qualitative inquiry from March to April 2015. Quantitative data were collected from 631 mother-infant pairs residing in Aysaita woreda with a pretested structured questionnaire using the modified expanded program on immunization cluster sampling procedure. Seven clusters were selected using probability proportional to size.

The qualitative data were generated through two focus group discussions among purposely selected discussants: one group of eight health professionals and another group of mothers, fathers and traditional birth attendants (*n* = 10). Bivariate and multivariable analysis was done using binary logistic regression model while thematic framework analysis was employed for the qualitative data.

**Results:**

The prevalence of EBF under six months of age was 340/618 (55%). Infants whose mothers resided in an urban area [Adjusted Odds Ratio (AOR) 5.7; 95% Confidence Interval (CI) 3.5, 9.2), were knowledgeable about breastfeeding (AOR: 2.3; 95% CI 1.6, 3.5) and delivered at health facilities (AOR: 1.7; 95% CI 1.1, 2.7), were more likely to be exclusively breastfed than the referent group. In addition, mothers had a poor understanding of what constitutes exclusive breastfeeding. Traditional beliefs, myths and misconceptions about EBF and lack of support from husband and family were found to be barriers for proper EBF practice.

**Conclusions:**

The prevalence of EBF did not meet the World Health Organization recommendations. Factors related to infrastructure, service delivery, health education packages and traditional beliefs were associated with EBF practice.

## Background

Global rates of exclusive breastfeeding have remained stagnant since 1990 with only 36% of children aged less than 6 months exclusively breastfed in 2012 and with only slight increment in year 2016 (i.e. 40%) of children less than 6 months exclusively breastfed [1].

In developing countries, there are approximately 56 million infants less than 6 months of age, approximately 22 million are exclusively breastfed, while over 34 million children are not [2]. Eighty percent of these children who do not benefit from exclusive breastfeeding in developing countries live only in 29 countries. From these 29 countries, the 10 large countries including Ethiopia have two-thirds (over 21 million) of the approximate numbers of non-exclusively breastfed children in developing countries [2].

In a pastoralist area such as Afar, one in five children suffers from acute malnutrition. The high rate of malnutrition contributes to the country’s elevated under-five mortality rate of 88 deaths per 1000 live births, with malnourishment accounting for over half of all under-five deaths. Prelacteal feeding, bottle feeding, discarding of the colostrum, and other cultural taboos play a significant role in poor caring and feeding practices. Health service coverage is limited while antenatal care in Afar (19.2%) is well below the national average (52.1%) [3].

The Afar region ranks top among all regions of Ethiopia regarding having the highest number of stunted and wasted children. According to the Ethiopian Demographic Health Survey (EDHS) 2011, the Afar region has the lowest exclusive breastfeeding median duration which is 0.6 months [4] next to Somali 0.5 months, which is again far beyond the national median duration of 2.3 months.

On top of that seasonal variances in food consumption, water availability, sanitation, and breastfeeding practices are poorly understood but contribute greatly to the prevalence of malnutrition in Ethiopia and particularly in Pastoralist areas such as Afar [3]. The main reason why there are such seasonal variation particularly in our study area is frequent changes in the climatic condition. These changes are often followed by drought [5].

Investigation of the prevalence and associated factors of EBF may provide insight into the current burden and nature of the problem and help on how to direct prevention strategies. Although several studies have been conducted on EBF in many regions of Ethiopia, the EDHS with limited factors remains the only study that was carried out on the specific area, which clearly suggests that there is a wide research gap on the study area. The aim of this study was to measure the prevalence and identify associated factors of exclusive breastfeeding practice in Afar.

## Methods

### Study design and population

A community based cross-sectional study with focus group discussion (FGD), i.e. with healthcare providers and parents, was conducted from March, 2015 to April, 2015. Mothers with infants under the age of 6 months from pastoral and agro-pastoral Ethiopia; Aysaita, were eligible to participate in the study. Aysaita is 655 km from Addis Ababa and 65 km from the capital city of Afar, Samara. According to the 2007 Census conducted by Central Statistical Agency (CSA) of Ethiopia; the total population of Aysaita was 55,519, whereas the total population of under five and under 1 year was 7645 and 1654 respectively and a total households (HH) of 9740. About 91.7% of the population lives in rural areas; pastorals and agro-pastoral system of livestock production is the dominant source of livelihood.

### Sample size determination and allocation

The required sample size was determined based on a prevalence rate of 52% of exclusive breastfeeding infants under 6 months of age from the national EDHS 2011; with 5% level of significance, 5% margin of error and 10% non-response rate; yielded 383. We used single and double population formula to accommodate the objectives of the study. The sample size was inflated with a design effect of 1.5 and the total sample size reached 631. Samples were allocated proportionally across clusters using the Expanded Program for Immunization (EPI) sampling technique.

### Sampling procedures

#### Quantitative sampling procedure

Aysaita woreda (woreda is a district and the third-level administrative division of Ethiopia) has a total of 9740 households (HH), 13 kebeles (wards or neighborhood associations, which are the smallest unit of local government in Ethiopia), two urban and the remaining 11 rural kebeles. The kebeles with their respective live infant population are; Kebele 02 (265), Kebele 01 (297), Kerebuda (100), Berga (79), Keredura (67), Mamula (109), Henele (99), Henedegi (111) Gaharetu (69), Galefagi (143), Romayity (81), Galealo (69) and Ehahile (164). To select the representative data this study aimed using modified EPI cluster sampling.

A selection of HHs within a community should ideally be random and in practice this is most closely achieved by systematic selection from a numbered list of HHs. In many situations like this specific research however there is no list or map for HHs available. We didn’t have a resource to completely enumerate or map all the HHs in the community and so some compromise must be used. As up to date information on the total estimated population was difficult to obtain and as simple random sampling was almost impossible to accommodate due to unavailability of list of all the HHs and a resource limitation, the investigators found the modified EPI cluster sampling scheme [6] to be a useful alternative approach. The modified EPI cluster sampling method is a type of cluster sampling developed by the WHO expanded program for immunization to estimate vaccination coverage. This procedure led to the selection of clusters and HHs.

### First stage sampling: Cluster selection

#### Population proportion to size (PPS)

The PPS sampling method led to selection of clusters/kebeles, so that each household had equal probability of being selected in the sample. This procedure is self-weighting. Accordingly, selected clusters were 02-kebele, 01-kebele, Kerebuda, Mamula, Henele, Galefagi and Ehahile.

### Second stage sampling

After selecting the seven clusters which were then were called enumeration areas (EA), the second stage to be carried out was a method on how much HHs to allocate for each EA’s to acquire the calculated sample size of 631. As all the clusters are selected proportional to their size, we found it appropriate to divide and distribute an equal number of HHs for each EA’s. Dividing 1958 HHs (estimated HHs to be visited) equal to the 7 EAs, we found a number, 280, and 280 HHs in each selected cluster or EAs were visited.

### Household (HH) selection

#### Selecting the first and the consecutive HHs

These involved two stages, a method of selecting the first household (HH) to be the starting point and a method of selecting the successive HH after that. To select the first HH we used the EPI recommendation for selection of the first HH. That was, we chose a central point in the village (a market), and then we chose random direction from that point, counted the number of HHs between the central point and the edge of the town and randomly selected one HH to be the starting point. The remaining HHs were selected in order of giving us a wide spread coverage consistent with practicality. According to the EPI strategy we went to the HH whose door was nearest to the door from the HH we stood up.

### Qualitative sampling procedure

Before conducting the focus group discussion (FGD) we did a situational analysis in order to help us on selection of our participants. The key informant was a case team leader for maternal and child health and a supervisor assigned for Aysaita woreda health bureau from the regional health bureau. He was a person in a position to know the healthcare providers in the woreda very well. The key informant suggested for one group of health professionals FGD, as it would be infeasible to make a number of professional groups. Because most professionals were working on the rural kebeles, it was found to be difficult to call-upon healthcare providers from all the kebeles and conduct a number of different group discussions. The reason was; if they were to come up to one location together it would make them leave their duties and one health post has a maximum of two health professionals, excluding HEW i.e. a health officer with/without one nurse or two nurses). It was suggested to call-upon the professionals that were found on the urban and the nearby rural kebeles, so that, we have tried to include different health professionals and limited our FGD to one.

The same was true for the other FGD. It was suggested to limit it to one FGD. Limited means for transportation to the rural kebeles and finding enough translators were the main challenges from the key informant location, which hindered us from conducted more FGDs. Accordingly, we were obliged to limit our sample selections from the mothers and fathers who came to the health facilities. We have conveniently selected mothers and fathers while they came to the health facilities (three health facilities) for healthcare treatment and we selected one traditional birth attendant with the suggestion of HEW (a participant on the healthcare professional FGD).

In order to minimize the possible bias that the key informant may have on selection of the participants, in our explanations we made sure to emphasize that we want a group of people that can express a range of views, to be able to have a proper discussion.

We have tried to create a smooth discussion environment and tried to encourage the communication and interaction during the FGD in every possible way we could.

- We have tried to hold the FGD in a neutral setting which encourages participants to freely express their views.
- We made sure that there were no disturbances, sufficient quietness, adequate lighting, adequate ventilation as it was the hottest season and also there were hot and cold beverages (liquid refreshments) including water.
- Materials that were necessary to conduct the FGD were prepared prior (i.e. FGD guide line, double voice recorders and note books, pen and pencils).
- We have also arranged the chairs in a circle so that we are able to create conducive discussion area.

#### Selection of participants for the healthcare providers FGD

The Aysaita Regional Health Bureau (ARHB) reported that the district has one hospital, one health center, 10 health posts, and four private clinics. Employees in the health centers and health posts were a total of 87; 32 were technical and the other 55 supportive. There was a total of 20 health extension workers in the woreda, and all 20 working in the rural setting. We made our selection from the HC and HPs and from the woreda health bureau. With the help of the of the key informant we selected eight healthcare professionals. Accordingly, we selected one administrator (public health expert with educational background), two nurses, two HOs and three HEW from Aysaita HC which is located in the urban and nearby rural HPs (Henele, Berga and Ghalefage).

#### Selection of participants for community FGD

We conveniently select a total of fiv5 mothers and 4 fathers from urban and nearby rural kebeles (2 mothers and 2 fathers from Kebele 1 & 2 and 3 mothers & 2 mothers from Henele kebele), while they came for healthcare treatment to Aysaita Health center and Henele Health post. Although we couldn’t be able to know the specific number of available traditional birth attendant, with the recommendation of one of the participants in the healthcare professional FGD, a health extension worker, we selected one traditional birth attendant.

### Data collection tools and procedure

Data were collected using a structured interviewer administered questionnaire and focus group discussion guide.

#### The questionnaire

A questionnaire with both closed and open-ended questions was used to collect information on infant characteristics (sex and age), maternal demographic characteristics (age, education and marital status), maternal socioeconomic characteristics (occupation income, and ownership of items), maternal attitude and knowledge on breastfeeding [7–10], sources of breastfeeding information, maternal delivery experience and infant feeding practices. The questionnaire was adopted from the EDHS, WHO and related studies. The questionnaires were initially prepared in English and then translated into Amharic and Afar. The Afar version was again translated back into Amharic and English to check for any inconsistencies or distortion in the meaning of words and concepts.

### Focus group discussion guide

A focus group discussion (FGD) guide was used to elicit information on infant feeding practices with special focus on knowledge, attitudes and beliefs about exclusive breastfeeding and factors influencing the practice of exclusive breastfeeding. This information was intended to provide an in-depth understanding of infant feeding practices as well as offer an understanding or explanation of the quantitative findings. The FGD included mothers/caregivers, fathers, grandparents, healthcare providers and traditional birth attendants and the group included both rural and urban participants. Two FGDs were conducted with 8 to 10 participants in each group. The primary investigator (PI) together with a translator took the primary lead in eliciting the questions and modulating the discussion.

### Operational definitions

*Exclusive breastfeeding*: Was measured using a single 24-h recall method where by mothers are asked if they fed their baby only with their breast milk (including milk expresses from a wet nurse), ORS, drops, syrups (vitamins, medicine and minerals) and nothing else in the last 24 h prior to the interview.

*Predominant breastfeeding*: Fed on breast milk (including milk expressed or from a wet nurse) certain liquids (water, water-based drinks fruit juices), rituals and ORS, drops or syrups (vitamins, medicine and minerals) as the predominant source of nourishment.

*Partly fed*: Breast milk (including milk expressed or from a wet nurse), any food or a liquid including non-human and solid or semi-solid foods milk and formula.

*Complementary breastfeeding*: Fed on breast milk and complementary foods (milk, porridge, semi-solids or solids).

*Non-breastfed:* Not fed on breast milk.

### Knowledge score about EBF

There were 10 questions (5 general questions and 5 maternal and child related questions) for assessing knowledge adopted from related studies. An average of responses on knowledge variables was done by computing variables and mothers who scored less than the average are labeled to have poor knowledge and those scored above as having good knowledge.

#### Attitude

Four scaled Likert scale was used to measure the opinions of mothers towards EBF. All attitude opinion variables were computed and averaged. Those scored below the average were considered with negative attitude and those scored above the average were considered with positive attitude.

### Data quality management

The quality of data was assured before, during and after data collection process.

#### Before data collection

Objective based and standardized designing of questionnaire, preparation of data collectors training manual, experience-based selection of data collectors and finally training of data collectors (10 in number) and one supervisor on sampling procedures, techniques of interviews and data collection process and giving of training manuals and actual training was performed. In addition, the data collectors and supervisors were participated in pre-testing of the questionnaire for its understandability by 5% of sample on volunteer individuals in kebeles which were not included in the actual data collection. The purpose of the pre-test was to ensure that the respondents be able to understand the questions and to check the wording, logic and skip order of the questions in a sensible way to the respondents. Amendments were made accordingly after the pretest.

#### During data collection

The supervisors and principal investigator were closely following the day-to-day data collection process and ensure completeness and consistency of questionnaire administered each day.

#### After data collection

The collected information was rechecked for its completeness and consistency by the supervisors and principal investigators before transferring in to computer software. Non-over lapping numerical code was given for each question and the coded data were entered and cleaned into SPSS software Version 21.

### Data analysis procedures

The collected data was checked for completeness and entered to SPSS version 21 statistical software for each specific objective step wise. According to the specific objectives, descriptive statistic including Proportion, frequency distribution, Mean (SD) and Median was used to describe the data on the sample population in relation to relevant variables.

Bivariate analysis; cross tabulations was done to see the association between the explanatory and outcome variables. Multivariate analysis; Binary logistic regression model was employed by selecting only variables that appeared to be statistically significant at (*p* < 0.05) in the bivariate analysis. A thematic content analysis was used for analyzing the qualitative data’s using Open code software application and data and environmental triangulation were done, which allows for different stake holders on exclusive breastfeeding (i.e. mothers, elders, fathers, traditional birth attendants and healthcare professionals) to participate and there by maintain the validity of the results. Also, the FGD included participants both from urban and rural areas so as to have a better understanding if residence has effects on proper EBF practice. Finally, the data was presented with appropriate tables, diagrams and figures.

**Household wealth** was assessed by constructing an index using Principal Component Analysis (PCA). The domains to construct the model include characteristics of the house including floor, wall, roof, type of toilet facility, source of water, ownership of agricultural land, ownership of an animal including goat, camel, sheep, chicken, donkey, ox and cow, ownership of fixed assets such as; motorcycle, car/truck, television, cell phone, phone, refrigerator, clock, bed and main source of fuel for cooking. Cut-off points were used to divide the data into three equal groups; low, medium and high, resulting in quintiles representing the wealth status of the households.

## Results

### Descriptive results

#### Socioeconomic and demographic determinants of the households

A total of 631 households were included in the study and 98% response was obtained from the respondents. The mean age of the infants was 3.6 (± 1.6 SD) months and 128 (20.7%) were 4 months old. Married respondents were 588 (95.1%) and 524 (84.80%) were Muslims. Three hundred seventy-five (60.7%) mothers were illiterate or with no formal education. Mothers who were employed were 120 (19.4%) (Table 1).

**Table 1:** Demographic and socioeconomic characteristics and early breastfeeding experience of the respondents Aysaita woreda, Afar, 2015

Variables	Categories	*n*	%
Residence	Urban	309	50
	Rural	309	50
Wealth	Low	199	32.3
	Middle	208	33.8
	High	209	33.9
Support	Yes	571	92.4
	No	47	7.6
Mothers age	15–25	296	47.9
	26–35	288	46.6
	36–45 & above	34	5.5
Marital status	Married	588	95.1
	Divorced	30	4.9
Educational status	Illiterate	375	60.7
	Primary	170	27.5
	Secondary level & above	73	11.8
Religion	Muslim	524	84.8
	Christian	94	15.2
Maternal employment	Employed	120	19.4
	Not employed	498	80.6
Parity	1	133	21.5
	2–4	390	63.1
	5+	95	15.4
Delivery place	Health facility	396	64.1
	Home/At TBA’s house	222	35.9
Delivery type	Spontaneous	598	96.8
	Cesarean	20	3.2
Child sex	Male	341	55.2
	Female	277	44.8
Child age	Up to 1 month	78	12.6
	2 months	83	13.4
	3 months	119	19.3
	4 months	128	20.7
	5 months	116	18.8
	6 months	93	15

#### Early breastfeeding experience of the respondents

The majority of the mothers (63%) had 2–4 pregnancies. Twenty one percent of mothers had just one child and only 15% had five or more children. Five hundred and ninety-eight (96.8%) had spontaneous deliveries and the rest a cesarean. Three hundred and ninety-six (64.1%) mothers delivered at health facilities (Table 1).

#### Knowledge and attitude of the respondents

Three hundred and ten (50.2%) mothers had good knowledge about EBF and 308 (49.8%) had poor knowledge. Four hundred and five (65.5%) mothers had positive attitude towards EBF and 207 (33.5%) had negative attitude.

#### Knowledge

Three hundred and ninety-three (63.6%) mothers correctly answered the right time to give breast milk after the birth was immediately after delivery. Five hundred and forty-one (87.5%), of mothers answered the right time to start complementary food was at six months. Nineteen (3.1%) mothers/caretakers answered only breast milk as recommended for liquids or foods recommended to infants under six months. Three hundred and forty-nine (56.5%) mothers answered all options as correct (Table 2).

**Table 2:** Knowledge of respondents about exclusive breastfeeding Aysaita woreda, Afar region, 2015

Variables	Categories	*n*	%
Right time to give breast milk after the child is delivered	Immediately	393	63.6
	Within an hour	159	25.7
	Between 1 h and 3 h	64	10.4
	From 3 to 7 h	1	.2
	From 1 day to a week	1	.2
Right thing to do with the colostrum	Discard	76	12.3
	Feed immediately	535	86.6
	Other (specify)	5	.8
	I don’t know	2	.3
Right time to start complementary foods	3 months or less	4	.6
	4 months	25	4
	5 months	39	6.3
	6 months	541	87.5
	7 months or above	3	.5
	I don’t know	4	.6
Liquids or foods recommended to child under 6 months	Plain Water	77	12.5
	Infant formula	105	17
	Tinned milk or other kind of animal milk	62	10
	Fruit juice/ Yoghurt	5	.8
	Only breast milk	19	3.1
	All	349	56.5
	I Don’t know	1	.2

#### Attitude

Four hundred and fifty-eight (74.1%) mothers strongly agreed that giving breast milk immediately after birth was important. Two hundred and thirty-two (37.5%) mothers strongly agreed that discarding the colostrum was not important. Three hundred fifty-six (57.6%) of mothers strongly disagreed that starting complementary foods at age 4 months was important (Table 3).

**Table 3:** Attitude of mothers towards exclusive breastfeeding, Aysaita woreda, Afar region, 2015

Variables	Categories	*n*	%
Giving breast milk immediately after birth is important	Strongly agree	458	74.1
	Agree	88	14.2
	Disagree	67	10.8
	Strongly Disagree	5	.8
Discarding the colostrum before giving breast milk is not important	Strongly Agree	232	37.5
	Agree	219	35.4
	Disagree	148	23.9
	Strongly disagree	19	3.1
Starting complementary food at age of 4 month or before is important	Strongly Agree	7	1.1
	Agree	36	5.8
	Disagree	218	35.3
	Strongly disagree	356	57.6

#### WHO infant and young child feeding (IYCF) indicators

**Fig. 1:**
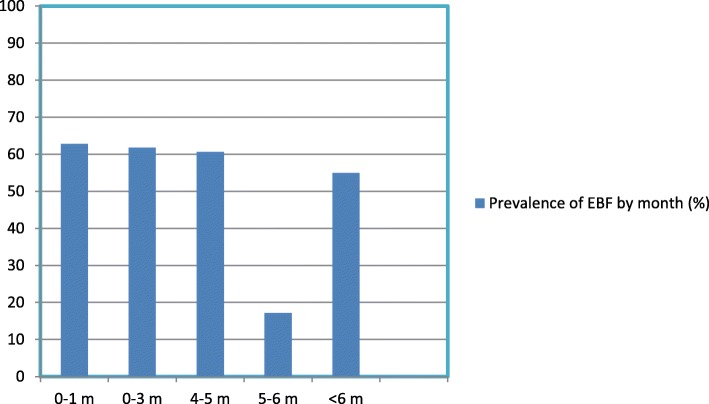
The prevalence of exclusive breastfeeding by month, Aysaita woreda, Afar Ethiopia 2015 by using 24-h recall

Almost all of mothers breastfed their infants. Three hundred and fifty (55%) of infants were exclusively breastfed. The month-specific prevalence of exclusive breastfeeding decreases as the age of the babies increases 62.8, 61.8 and 60.6%; at age < 1 month (i.e. babies in the age range of 0–29 days), 0–3 months (babies in the age range of 0–89 days) and 4–5 months (i.e. babies in the age range of 120–149 days) respectively. The rate dramatically declines to 17.2% at age range 5–6 months (i.e. babies in the age range of 150–179 days) (Fig. 1). One hundred and twenty (19.4%) of children were predominantly breastfeeding. Two hundred and twenty-five (36.4%) of mothers practiced prelacteal feeding (Table 4).

**Table 4:** IYCF indicators, Aysaita woreda, Afar region, 2015

Variables	Categories	EBF	*n*	%
Exclusive breastfeeding (% *n*)	0–1 months	Yes	49	62.80
		No	29	37.20
	2–3 months	Yes	127	62.90
		No	75	37.10
	0–3 months	Yes	176	62.90
		No	104	37.10
	4–5 months	Yes	148	60.40
		No	97	39.60
	5–6 months	Yes	16	17.2
		No	77	82.8
	< 6 months	Yes	340	55.00
		No	278	45.00
Predominant breastfeeding	Yes		120	19.4
Early initiation of breastfeeding	Yes		496	80.30
Children ever breastfed	Yes		605	97.90
Prelacteal feeding	Yes		225	36.40

### Bivariate analysis

#### Factors associated with breastfeeding

In the bivariate analysis, residence, maternal educational status, marital status, religion, maternal age, parity and knowledge about EBF were found to be associated with practice of exclusive breastfeeding. Children of mothers/caretakers who reside in urban areas were seven times more likely to be exclusively breastfeed with crude odds ratio (COR): 7.0; 95% CI 4.9, 10.0 compared to children of mothers who reside in the rural areas. Children of mothers who have primary, secondary and greater education were more likely to exclusively breastfeed compared to children of mothers who were illiterate/had no education; COR: 1.7; 95% CI 1.2, 2.5 and COR: 2.4; 95% CI 1.4, 4.1 respectively. Children of mothers who delivered at health facilities were four times more likely to be exclusively breastfed, COR: 4.4; 95% 3.1, 6.2 compared to children of mothers who delivered at home/traditional birth attendant (TBAs) home. Children of mothers who have good knowledge on the practices of EBF were 3.6 times more likely to be exclusively breastfed, COR: 3.6; 95% CI 2.6, 5.0 compared to children of mothers who have poor knowledge on practices of exclusive breastfeeding. Children of mothers who have 1 and 2–4 pregnancies were around four times more likely to be exclusively breastfed, COR:3.9; 95% CI 2.2, 6.9 compared to mothers who have five or more pregnancies (Table 5).

**Table 5:** Factors associated with exclusive breastfeeding, Aysaita woreda, Afar region, Ethiopia, 2015: Bivariate analysis

Variables	Categories	Exclusive Breastfeeding				*p* - value	Crude odds ratio (95% CI)
		Yes		No			
		*n*	%	*n*	%		
Total		340	55	278	45		
Residence	Urban	239	77.3	70	22.7	0.00	7.0 (4.9,10.0)
	Rural	101	32.3	208	67.3		1
Educational status	Illiterate	183	48.8	192	51.2		1
	Primary	106	62.4	64	37.6	0.003	1.7 (1.2,2.5)
	Secondary & Above	51	69.9	22	30.1	0.001	2.3 (1.4,4.2)
Knowledge	Good	212	70.3	92	29.7	0.00	3.6 (2.6,5.0)
	Poor	122	39.6	186	60.4		1
Employment	Employed	61	50.8	59	49.2	0.305	0.8 (0.5,1.2)
	Not employed	279	56	219	44		1
Delivery place	Health facility	268	67.7	128	32.3	0.00	4.4 (3.1,6.2)
	Home/At TBAs house	72	32.4	150	67.6		1
Parity	1	81	60.9	52	39.1	0.00	3.9 (2.2,6.9)
	4–5	232	59.5	158	40.5	0.00	3.7 (2.2,6.0)
	5+	27	28.4	68	71.6		1
Mothers age	15–25	151	51	145	49	0.43	2.2 (1.0,4.6)
	26–35	178	61.8	110	38.2	0.02	3.4 (1.6,7.2)
	36–45 & above	11	32.4	23	67.6		1
Marital status	Married	318	54.1	270	45.9		1
	Divorced	22	73.3	8	26.7	0.44	2.3 (1.0,5.3)
Religion	Muslim	273	52.1	251	47.9		1
	Christian	67	71.3	27	28.7	0.001	2.3 (1.4,3.7)

### Multivariate analysis

#### Factors associated with exclusive breastfeeding

In the bivariate analysis any possible confounders were not controlled and assessing the independent effects of the covariates was difficult. So, enter method of logistic regression technique was used to assess the independent effect of explanatory variables on exclusive breastfeeding. To avoid excessive number of variables and unstable estimate in the final model, only variables with *p* - value less than 0.05 in the bivariate analysis were taken in the multivariate analysis. Model fit was checked by Hosmer-Lemeshow goodness-of-fit test.

The multivariate logistic regression analysis identified urban residence, primary educational status of the mother, low parity, delivering at health facilities and having a good knowledge about EBF as factors associated with exclusive breastfeeding.

Children of mothers who reside in urban areas were around six times more likely to be exclusively breastfed (Adjusted Odds Ratio [AOR]: 5.7; 95% CI 3.5, 9.2) compared to children of mothers who resides in rural areas. Children’s of mothers who delivered at health facilities were 1.7 times more likely to be exclusively breastfed (AOR: 1.7; 95% CI 1.1, 2.7) compared to children of mothers who delivered at home/TBAs home. Children of mothers who have good knowledge on the practices of EBF were two times more likely to be exclusively breastfed (AOR: 2.3; 95% CI 1.6, 3.5) compared to children of mothers who have poor knowledge on practices of EBF. Children’s of mothers who have 1 and 2–4 pregnancies were about twice as likely to be exclusively breastfed (AOR: 2.2; 95% CI 1.2, 4.2 and AOR: 1.9; 95% CI 1.1, 3.5 respectively) compared to children of mothers who have more than five pregnancies (Table 6).

**Table 6:** Factors associated with exclusive breastfeeding in Aysaita woreda, Afar region, Ethiopia, 2015: Multivariate analysis

Variables	Categories	Exclusive Breastfeeding				Crude Odds ratio (95% CI)	Adjusted odds ratio (95% CI)
		Yes		No			
		*n*	%	*n*	%		
Total		340	55	278	45		
Residence	Urban	239	77.3	70	22.7	7.0 (4.9,10.0)**	5.7 (3.5,9.2)**
	Rural	101	32.3	208	67.3	1	–
Educational status	Illiterate	183	48.8	192	51.2	1	–
	Primary	106	62.4	64	37.6	1.7 (1.2,2.5)*	0.6 (0.4,0.9)*
	Secondary & Above	51	69.9	22	30.1	2.4 (1.5,4.2)*	0.6 (0.3,0.9)
Knowledge	Good	212	70.3	92	29.7	3.613 (2.587,5.045)**	2.3 (1.6–3.5)**
	Poor	122	39.6	186	60.4	1	–
Delivery place	Health facility	268	67.7	128	32.3	4.4 (3.1,6.2)**	1.7 (1.1,2.7)*
	Home/At TBAs house	72	32.4	150	67.6	1	–
Parity	1	81	60.9	52	39.1	3.9 (2.3,6.9)**	2.2 (1.2,4.2)*
	“2–4”	232	59.5	158	40.5	3.7 (2.267,6.034)**	1.9((1.1,3.5)*
	5+	27	28.4	68	71.6	1	–
Mothers age	15–25	151	51	145	49	2.2 (1.0,4.7)	1.3 (0.6,3.5)
	26–35	178	61.8	110	38.2	3.1 (1.6,7.2)	1.5 (0.6,3.7)
	36–45 & above	11	32.4	23	67.6	1	–

*p - value < 0.05 **p – value < 0.01

### Focus group discussion results

We had two FGDs. One with health professionals including health administration, nurses, health extension workers and health officers who worked in Aysaita regional health bureau, health centers and health posts from selected urban and rural kebeles of Aysaita. The other FGD was with mothers (young and elder), fathers from rural and urban areas and one traditional birth attendant.

The results are presented in two major themes and sub themes. Theme one being community’s knowledge about EBF and the other theme factors (barriers) for EBF practice.

### Knowledge about exclusive breastfeeding

#### Poor understanding of EBF

This study has revealed that mothers did not understand what constitutes EBF (i.e. what it means and for how long it is recommended). For the majority of mothers, the idea of giving only breast milk to a new born was surprising and unusual, as long as they did not give solid foods, they considered themselves having practiced EBF even if they gave infants water, milk, fruits and biscuits. They do not differentiate EBF from partial and predominant breastfeeding as can be seen in the following quote:

*“*. *.*. *A women is obliged to breastfed her baby until at least 2 years and it’s our culture. I gave all my children water, goat and cow milk mixed with water and sometimes biscuits soaked with milk*. *.*. *I didn’t give solid foods like bread or ‘enjera’ and I believe with my breast milk this is the right way to raise my only 3 month old baby.”* Mother

Giving infants butter, water and milk is a common practice by mothers in the study area. Infants are given water as early as immediately after birth while others are given water during that period before the mother starts producing milk. It is believed that babies need water to quench thirst as the area is one of the hottest areas in Ethiopia. They also gave water to sooth them when they cry, especially during the absence of their mothers. Giving milk is also other common practice, as the majority of households depend on livestock for their livelihood, and most of their consumption is milk and meat they believe it will give the baby an early chance to get used consuming food and drinking the milk.

*“Our land as you see is too hot. The baby needs cold water to quench his thirst and we give them immediately after birth and as often as possible we think the baby needs.”* Elder mother

*“I gave milk and water when his mother is a way to stop him from crying. We gave them especially milk so as they have chance to get used to our living style, as Afars milk is our daily food, we also believe milk is very important for the growth of the child”* Elder mother

*“*. *.*. *the moment the child is born we nourish him with a drop of water, we care for his body with a smoke of different aromas as to our culture*. *.*.” Elder mother

### Factors (barriers) for EBF practice

#### Traditional beliefs, myths, and misconceptions about breastfeeding

This study found that mothers give their infants “fresh” cow or goat butter, milk or water to swallow immediately after birth in fulfilling one of traditional practices called *“turufa/‘onn’orr/ferenga’etu”*, where a relative, friend or anyone who is considered influential (i.e. religious man or woman, hero from previous war(s), a person from rich family, great personality. .. etc.) by the family and or the community is selected and give fresh cow or goat butter, milk or water to the infant in believe that the infant will gain the characters of the person who is giving the butter, milk or water. The process is called to do *turufa* or *onn’orr* and the person is called the babies *ferenga’etu* and vice versa.

*“I had my best friend who have my mother’s name, and loved by my mother, that my mother is her “Ferenga’etu.” When I was pregnant, I told my best friend that I will give her my child for onn’orr if she is a girl. It was a girl and my best friend have gave her goat butter immediately after she is delivered and that allows us even more loving best friends and I believe she will be good religious person as my best friend and you can see all the great characters and love as she grow up*. *.*.” Mother

This practice of giving a baby butter, milk and sometimes water is reinforced by important and influential members of the family, such as grandmothers and mothers-in-law, fathers, religious leader as told by the traditional birth attendant.

*“Turufa/onn’orr is our culture and we believe it should continue as we didn’t see any harm except our babies became as a person we want them to be”.* Elder mother

Health professionals who participated in this study acknowledged the existence of these beliefs, myths, and misconceptions about breastfeeding. According to health workers, these beliefs are a result of a lack of knowledge and understanding of breastfeeding by mothers and the community at large.

*Ahmed, nurse stated “The community has a lot traditional practice towards child nutrition “turufa/ferenga’etu.” is one of it where by mothers select a good character relative or friend to give the infant fresh butter and other fluids in belief that their child will get the good character of that person”*

#### Insufficient breast milk production

Perceived insufficient breast milk production emerged as one of the barriers to EBF. This perception is one reason why mothers introduced their babies to other foods earlier than recommended. Baby’s weight, age, crying during or after breastfeeding and the hot weather are found to be important indicators by which mothers tell that the baby is not getting enough nutrients from the breast. As a solution, grandmothers and other community elders advise the mother to introduce the baby to more milk, biscuits, bread soaked in tea, thin porridge and other complementary foods often much earlier than the recommended time:

*“The babies are too small to survive only with breast milk. I advise my daughter and also mothers in my neighborhood to give them lite foods specially if they are crying and if their weight is same time to time or if they are not gaining weight, as the babies grows they need more solid foods. Also they cannot stand the hot weather without additional foods and fluids as they sweat and all the fluids evaporate by the heat”* Traditional birth attendant

*“I start to give my child a cow milk at age of 3 months because I believe that only my breast milk was not enough and I was afraid my baby will get hungry”* Mother

#### Lack of support from husband and family

Lack of support from family members, especially husbands, is valued by mothers and health professionals as an important barrier factor for a breastfeeding mother. Men provide not only physical needs, emotional and psychosocial support for the mother but also financial support thereby creating a stable economy and an enabling environment for a mother to practice exclusive breastfeeding.

#### Husbands as source of income

This study has revealed that the majority mothers are dependent on their husband for income. Therefore, particularly mothers who are divorced have a lot difficulty to support the child as most of the income came from the husband. They try to work as soon as possible after delivery (most of the time home to home laundry, cooking, day laboring in construction, etc.) and so it will be difficult to exclusively breastfed their child (i.e. as they move here and there leaving their child at home with bottle of milk, and even if they have their child with them they will carry bottle of milk and other solid foods with them and they feed them the foods and fluids together with the breast milk). The other reason is they will face is an influence from the community on the whole child bearing process from the community (i.e. mothers, mother in laws, elders, neighbors, etc.) the community try to share the burden of supporting the single mother and in order to continue to get the support the mother accept everything they say, which includes how to feed, bath, etc. the child and most of the elders believe in giving extra foods and liquids to the infant and she just accept.

*“I have divorced my husband while I was pregnant with my 3*^*rd*^ *baby, I didn’t have any education or business to support myself and my family, so I have to live my 2 babies with my mother in low as mine were elders and I went out to work as a laundry woman. I used to wash cloths moving home to home by my hands and earn some money. After around 2 months of my delivery again I have got continue to work washing clothes home to home*. *.*. *sometimes I took her with me carrying bottle of milk and biscuit other times I leave her with my neighbors with milk and other light foods”*

#### Husbands sharing jobs

This study revealed that mothers in both rural and urban areas carry almost all the burden of the household work. As echoed on the discussion from the health workers, fathers and mothers traditional work is shared differently between men and women, i.e. men do certain jobs and so do women. Women share most of the work in the household or we can say women share all the household work (cooking, fetching water, laundry, taking care of the babies) and husbands are left to take care of the animals (move with the animals to find food and water for the cattle, camels and goats) in rural areas, and an office job or business in urban areas.

*“My husband left me for 3 months moving with the camels for food and water to faraway villages where he can get a water and grass for our camels*. *.*. *I was left alone with my elder mother home and I have to do everything by myself*. *.*. *I used to give thin porridge made from corn to my baby, goat milk and water as I am busy and couldn’t be able to breastfeed him as I want to.”* Mother

Other close and important family members, such as younger sisters, grandmothers and mothers-in-law, provide the necessary encouragement that mothers need to continue breastfeeding, especially during times when mothers contemplate stopping breastfeeding:

*“Both my mother and mother-in law were with me and help me in breastfeeding my baby by supporting me in cooking and other housework and encouraging me to breastfed him as often as possible telling me that it is important to breastfed him longer in time – I wanted to stop.”*

#### Insufficient healthcare providers and lack of EBF in the health education packages

This study has revealed that the regional health bureau has initiations and works ongoing regarding maternal and child health and nutrition. The health professionals acknowledged that the community has limited knowledge on child feeding in general and specifically exclusive breastfeeding and they are working to address the gap. One of the strategies is providing health education using health extension workers both in rural and urban areas, where by the HEW reach to the villages and provide the education to the mothers.

*“Other than doctors, nurses and health officers we have health extension workers who are trained in different health education packages (16) including child nutrition, they reach the community and provide health education, encourage mothers to vaccinate their children and notify mothers on the date of their children’s vaccinations.”* Health professional

As we have tried to look on the packages of the education/messages that are delivered, we have found that most are concentrating on reproductive health and vaccination.

*“Besides the health extension package, for example we have one project which we started 3 months before collaborating with UNICEF, where we train mothers from each kebele about family planning and women reproductive health and assign these women as committee head in their village and let these women train all mothers grouping them in 1 to 5 in selected topics every 15 days. Discussion topics are picked after the end of each discussion as raised by the mothers and most of the time it’s on reproductive health issues. After the discussion they report to the health facility (health post or health center) and gain feedback on the issues”* Health professional

Mothers from the rural areas also confirmed that health extension workers come and give them education on health-related matters specially vaccination. Mothers from the urban areas said that health extension workers are not available on the urban areas and they have an access for child feeding counseling during their visit for ANC and post-delivery.

*“I believe it would be better if we have more health extension workers in the rural areas as the villages are dispersed and health facilities are not in every village and as the health extension workers can only reach the nearby villages. Because I believe health extension workers brought a remarkable attitude changes toward many health problems in the community specially vaccination and ANC follow up and HIV/AIDS*.” Father and health extension worker

## Discussion

This study attempted to determine the prevalence of EBF, knowledge about EBF practice, attitude towards EBF and associated factors of exclusive breastfeeding practice. The prevalence of ever breastfeeding was 97.6% while the crude prevalence of exclusive breastfeeding for infants under 6 months of age was 55%. In the multivariate analysis, urban residence, good knowledge about EBF, delivering at health facilities and low parity were found to be associated factors for exclusive breastfeeding among the infants aged less than 6 months.

The majority of mothers (97.6%) breastfed their babies. This result is comparable to the EDHS 2011 [4] whereby the rate of infants ever been breastfed in the country was 97.5 and 97.7% for Afar region, respectively. The prevalence of other regional states in Ethiopia ranges from 93.4% in Addis Ababa to 99.1% in Dire Dawa which was comparable with this study finding. The qualitative study also reveals that breast feeding is a common practice in the community, one of the TBAs stated that ‘*a women is obliged to breastfed her baby until at least 2 years and it’s our culture’*.

Although the World Health Organization (WHO), global and national infant and young child feeding guidelines recommend that all newborns should be exclusively breastfed their infants for the first 6 months [11], the crude prevalence of exclusive breastfeeding in this study was 55%. This was lower than studies done in Jimma town (60.1%), Goba district (71.3%), Ghana (64%), Nepal (66.6%) [12–15] and while higher than the finding from EDHS 2011, Arjo woreda, Jimma, Bahirdar [4, 7, 16] whereby the prevalence of exclusive breastfeeding were 47.9, 52, and 49.1% respectively. This difference is expected as the residents in our study area are part of a pastoral community. The study area is in a different setting than Bahirdar or Ghana where people are bound by number of traditional practices in day-to-day life and appear to be closed to evidence based facts. Also, as the study area is one of the regions where infrastructure such as electricity and roads is still under development, unlike Bahirdar and Ghana. Electronic based information such as advertisements, NEWS, announcements from the ministry of health or the regional health bureau might not be accessed. Also, availability to health services will be less which again hinders access to services like counseling, contributing to the low prevalence of exclusive breastfeeding. The high numbers are attributed may be because it’s a norm and a tradition to breastfed the babies in the community of Afar and that might contribute to the relative high prevalence of exclusive breastfeeding.

The month-specific prevalence of exclusive breastfeeding decreases as the age of the babies increases 62.8, 61.8 60.6% at age < 1 month (babies in the age range of 0–29 days, 0–3 months (babies in the age range of 0–89 days) and 4–5 months (babies in the age range of 120–149 days) respectively. In the age range of 5–6 month (i.e. babies in the age range of 150–179 days) the prevalence dramatically drops to 17.2% (See Fig. 1). This finding was more or less comparable with a study finding in Jimma where by the prevalence was 67.2, 24.3 and 8.4% at age ≤ 2 months, 3–4 months and > 4 months, Nepal [15] which showed that the prevalence of exclusive breastfeeding were 74, 24% and EDHS, 2011 [4] 70, 55, and 32% at 1 month, 3 months and above 4 months, respectively. As infants grew older and older, the prevalence of exclusive breastfeeding decreases significantly indicating the overall lower duration of exclusive breastfeeding in the study community. This is common in many developing countries as majority of mothers believe that breast milk alone was not sufficient as the age of infants grew older, mothers might have introduced complementary feeding for their infants due to the assumption that breast milk alone would not satisfy their needs as the infants are already older. Most of the mothers in the FGD described that they started to give their children cow milk at age of 3 months because they believe that only their breast milk was not enough. Traditional birth attendants also noted the babies are too small to survive only with breast milk and they advise mothers to give them light food especially if they are crying and if they are not gaining weight*.*

The possible explanation for the relatively high prevalence of EBF might be due to the fact that postpartum care is traditionally given in the first few months after birth where mothers remain at home, creating a chance to exclusively breastfeed their infant.

This study found a significant difference between mothers who resided in urban areas versus rural areas with regard to exclusive breastfeeding. Children of mothers who reside in urban areas were found to positively associated with EBF. This result is in line with studies done in Ghana, Awi, and Malawi [14, 17–19]. Many different reasons might explain these results. One likely reason might be that mothers who resided in urban areas might have better access to health facilities where they can take advantage of appropriate counseling and care for EBF. Another reason is that it is known that mothers in Afar have a lot of traditional practices concerning child care as one of the TBA witnessed the moment the child is born they nourish the baby with a drop of water*.* Traditional practices are often less prevalent in urban areas than rural areas possibly because the additional exposure and access to health, services and information through different media in urban areas. This access could be influencing women in urban areas to adopt new practices that maintain exclusive breastfeeding. Other possible reason would be, people in rural areas live a life characterized by lots of hardships due to lack of infrastructures, that led them to different works that requires physical strengths, and less access to services such as clean tap water, road, health service, electricity unlike the urban residents [15]. This might leave mothers to have less time to exclusively breastfeed their child as they share most of the responsibility for home works (i.e. fetching water, cleaning, cooking, taking care of the baby and etc.).

The majority of mothers in FGD also identified their husbands left them alone moving with the camels for food and water to a remote village where they can get a water and grass for camels and they have to do everything by themselves and couldn’t be able to breastfeed their babies as they want to.

Lack of nearby health facilities and poor road infrastructure makes it challenging for mothers in pastoral communities to travel to seek out the counseling and health education services about infant feeding that are delivered through these facilities. This is also supported by our FGD. Fathers and health extension workers reported that there should be more health extension workers in the rural areas as the villages are dispersed and health facilities are not in every village and as the health extension workers can only reach in the nearby villages*.*

In the current study, knowledge of mothers on Exclusive breastfeeding was 50.2% which is comparable to the study done in Jimma town (53.7%) but lower than the study finding in Malaysia (74.8%) [20]. This difference might be attributed due to the difference in awareness level about breastfeeding practice. Qualitative finding also indicted that even though health information dissemination is one of the targets of the ARHB and a work in progress there are still mothers who don’t have sufficient knowledge about EBF.

Being knowledgeable on the right time to initiate breastfeeding and complementary fluids and foods was positively associated with EBF. This finding was in line with many studies; Jimma, Taiwan, Tanzania [12, 21, 22]. This result is expected and the general explanation will be that the significance of having good and bad knowledge expected to have a direct effect towards a practice and the ARHB should continue to work on policies that encourage the availability of knowledge.

This study found that 66.2% showed favorable/positive attitude towards exclusive breastfeeding which was consistent with the study finding in Jimma town (73.9%) had positive attitude towards breastfeeding practice [12]. Although, in this study the attitude towards breastfeeding was generally favorable, > 24% respondents agree on discarding the colostrum before giving the first breast milk. This is probably due to insufficient knowledge on the issue, and attitude towards EBF is not associated in the bivariate analysis.

Having one, and two to four pregnancies was found to be associated with EBF, which is consistent with a study done in Jimma, whereby mothers having two and below children were more likely to practice exclusive breastfeeding.

Furthermore, the study found that a higher proportion of women who delivered at a government health facility exclusively breastfed compared to women who delivered at their own home or the home of the TBA. Place of delivery has been found in a number of studies to be associated with exclusive breastfeeding. In the present study, delivery at a government health facility was identified to be associated with exclusive breastfeeding and this conforms to studies in Ghana [14]. The association between delivery at hospital and exclusive breastfeeding can be attributed to the call made by WHO and UNICEF [11, 23] for hospitals to be centers of breastfeeding. This initiative, undoubtedly, might have accounted for government health facility being a predictor of exclusive breastfeeding in the country even though some health facilities may not have fully implemented the WHO recommendations. The findings of this study contrasted those of a study done in Nepal [15], where mothers who gave birth at home were more likely to practice exclusive breastfeeding. This is probably due to the fact that in Nepal, mothers who gave birth within health facilities are those with higher income and education, as health facilities are not equally accessible to all mothers. The situation is different in Afar, as public health facilities are relatively highly accessible for all regardless of income and educational status.

### Limitations and strengths

This study can be interpreted in light of its strengths and limitations. The use of validated questionnaires, both quantitative and qualitative methods of data collection and data triangulation, the fact that this study did assess individual factors; including knowledge and attitude of mother, as well as variables related to families and choosing and accommodating the study in remote and pastoralist area, Afar, rarely chosen place by researchers of any stream can be consider as major strengths of this study.

However, the 24-h recall to determine exclusive breastfeeding practice, an assessment method in which some infants who were given other liquids regularly may not have received them in the last 24 h before the survey, may have overestimated of the proportion exclusively breastfed. Many studies have shown that a large proportion of infants who were exclusively breastfed in the previous 24 h were either not exclusively breastfed during the previous 7 days, and/or, not exclusively breastfed since birth [24, 25].

Other factors like infant’s birthweight, antenatal care follow up, health status of the mother and the child and other factors which might be associated with exclusive breastfeeding were not addressed in this study. In addition, the lack of published articles on breastfeeding practices in pastoral areas could be mentioned as a limitation. Furthermore, this study used a cross-sectional study design, which made it difficult to establish causal effect relationship.

Limiting the qualitative study, due to various reasons, only having two focus group discussion can also add to the limitation of this study.

## Conclusions

Even though the crude prevalence of infants younger than 6 months who were ever breastfed was high, the prevalence of exclusive breastfeeding did not meet the WHO recommendation. In the current study, however half of the mothers had good knowledge about exclusive breastfeeding, knowledge of mothers about the right time to initiate breastfeeding after birth, and knowledge about foods/liquids recommended to infants less than 6 months was far lower in many studies done in developing countries. Most of the study population had positive/favorable attitudes towards exclusive breastfeeding practice during the first 6 months. Urban residence, having a good knowledge about EBF practices, low parity and delivering at health facilities were found to be associated with exclusive breastfeeding practice in the studied community. In addition, the qualitative inquiry revealed that mothers have a poor understanding of what constitutes EBF (i.e. what it means and for how long it is recommended). It is also revealed that traditional beliefs, myths and misconceptions about exclusive breastfeeding, perceived insufficient breast milk production, lack of support from husband and family*,* mother’s dependency on husband’s income, mothers having high burden of work in the home, inadequate number of healthcare providers and lack of properly addressing EBF in the health education packages for mothers, are all found to be barrier factors for proper EBF practice.

Thus strengthening efforts to enhance the infrastructures (i.e. road; so that health facilities are easily accessible for antenatal visits, delivery and counseling, electricity; for simpler life style and for easy information access through media, tap water; so that it will not take longer time for women to fetch water, health facilities; as health facilities are not still available for most of the rural villages and the dispersed placement of the pastoral community), promoting health education that are more specific on the importance and proper EBF practice; using accessible means such as medias and public meetings, elder, religious and influential persons, revising health education packages contents and delivery modalities, encouraging mothers to deliver at health facilities, creating income generating mechanisms for mothers to support themselves and their families; specially work on education strategies to combat the traditional beliefs, myths and misconceptions about EBF; such as apply trials to replace the traditional practice in a way that is not interfering with proper EBF practice, train more healthcare providers and revise means to reach the community, such as mobile clinics as the pastorals move from place to place and encouraging further research on the topic of area with objectives that will help on exploring on ways to are recommend for hand express milk and or explore to distribute the workload of the household are recommended.

## Abbreviations

AOR: Adjusted Odds Ratio
ARHB: Afar Regional Health Bureau
EAs: Enumeration Areas
EBF: Exclusive Breastfeeding
EDHS: Ethiopian Demographic and Health Survey
IYCF: Infant and Young Child Feeding
